# Diagnosis, treatment, and prognosis of scalp angiosarcoma: A case report

**DOI:** 10.1097/MD.0000000000046847

**Published:** 2026-01-09

**Authors:** Xuefeng Fu, Lei Zeng, Qiying Zhang, Yuxi Zhang

**Affiliations:** a Department of Dermatology, Jinhua Municipal Central Hospital Medical Group, Jinhua, Zhejiang, China; b Affiliated Jinhua Hospital, Zhejiang University School of Medicine, Jinhua, Zhejiang, China.

**Keywords:** angiosarcoma, case report, malignant tumor, scalp, treatment

## Abstract

**Rationale:**

Scalp angiosarcoma is a rare malignant vascular tumor with the characteristics of occult early symptoms, no specificity, a high misdiagnosis rate, strong invasion, and poor prognosis. The 5-year survival rate is <30%. The accumulation of rare cases is of great significance for optimizing diagnosis and treatment.

**Patient concerns:**

A 78-year-old man presented with a dark-brown nodule on the top of the head 2 months ago, which progressively enlarged with ulceration, erosion, and easy bleeding. Topical antibiotic therapy was ineffective. Imaging showed a mass with an unclear inferior boundary and rich blood flow (without bone destruction). Dermoscopy showed invasive growth with abnormal blood vessels. The diagnosis was confirmed by pathology and immunohistochemistry (positive for CD31, CD34, and ERG).

**Diagnoses:**

Angiosarcoma of the scalp was confirmed based on skin pathology and immunohistochemical results.

**Interventions:**

The scalp tumor was surgically removed under general anesthesia, and skin from the thigh was taken for transplantation.

**Outcomes:**

Skin necrosis and skull exposure in the graft area after the operation. The patient refused further repair treatment, imaging evaluation, radiotherapy, and chemotherapy, and died of massive hemoptysis 5 months after surgery.

**Lessons:**

Early identification and standardized comprehensive treatment are the core to improving prognosis. In clinical practice, it is necessary to accumulate rare cases, improve disease cognition, optimize diagnosis and treatment processes, and strengthen treatment compliance management to reduce mortality and improve quality of life.

## 1. Introduction

Angiosarcoma is a rare and highly malignant tumor of endothelial origin, accounting for about 1% of all soft tissue sarcomas. Due to its heterogeneity, it can involve multiple parts or organs of the body. It often occurs in the head and neck of the elderly over 60 years old, especially on the scalp.^[1–3]^ At present, the etiology of angiosarcoma is not clear, and it may be related to long-term ultraviolet exposure, chronic lymphedema, or a history of previous radiotherapy.^[4–6]^ Due to the strong invasiveness of angiosarcoma, it is easy to metastasize organs such as the lung and liver through the blood in the early stage, so the prognosis of patients is extremely poor, the 5-year survival rate is <30%, and even more than half of the patients die within 1 year of diagnosis.^[7,8]^ At present, the internationally recognized treatment for localized cutaneous angiosarcoma is still local surgical resection with negative margins and postoperative radiotherapy or adjuvant chemotherapy.^[9,10]^ This article describes a 78-year-old man with dark-brown nodules on the scalp that gradually enlarged, ruptured, and bled. He was diagnosed with scalp angiosarcoma by pathology and immunohistochemistry. The diagnosis, treatment, and prognosis can provide reference for clinicians.

## 2. Case presentation

**Figure 1.**
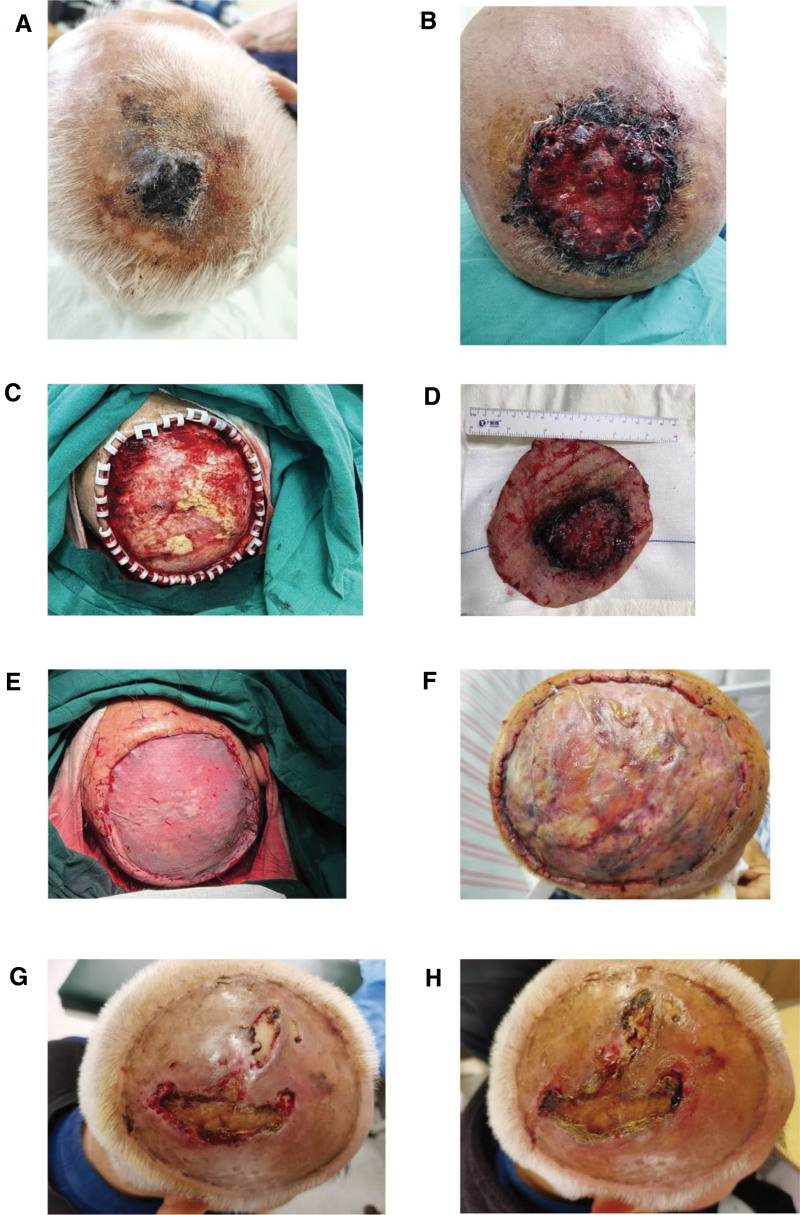
(A) The skin lesion at the patient’s first visit. (B) The patient’s skin lesions at the time of the current presentation. (C) During the patient’s operation, the scalp mass was widely removed. (D) Scalp mass (including erythema surrounding the mass) that was removed during surgery. (E) The skin from the thigh was used to graft the scalp defect. (F) Patient’s scalp situation on the third postoperative day. (G) Patient’s scalp condition 1 month after surgery. (H) The patient’s scalp condition at 3 months after surgery.

The patient was a 78-year-old man. He came to the hospital with a 2-month history of dark-brown nodules on his scalp. The patient had initially presented to the hospital when the smooth, dark-brown nodule on the scalp was noted (Fig. 1A). The clinic considered hemangioma and recommended resection, which the patient declined. During the next 2 months, the dark-brown nodules on her scalp rapidly became larger, and symptoms such as ulceration and bleeding occurred. He came back to the outpatient clinic again. According to the clinical symptoms, the doctor suggested that he undergo a biopsy. The patient refused again, and the doctor prescribed him an antibiotic ointment for external use. Due to the poor effect, he went to the outpatient clinic again, and the local skin pathological examination was performed this time, which showed angiosarcoma, so he was admitted to the hospital for further treatment (Fig. 1B). The patient, a former office worker, had no family history of cancer or radiation therapy, and had hypertension, coronary atherosclerotic heart disease, and diabetes for many years.

The patient’s heart and lung auscultation were normal on admission, and the superficial lymph nodes were not palpable. A brown mass with a size of 8 × 6 cm was found on the top of the head, with large black blood scabs on the surface and exudation of blood. There was a little erythema at the edge of the lesion (Fig. 1B). The rest of the hair grew normally.

### 2.1. Laboratory tests

**Figure 2.**
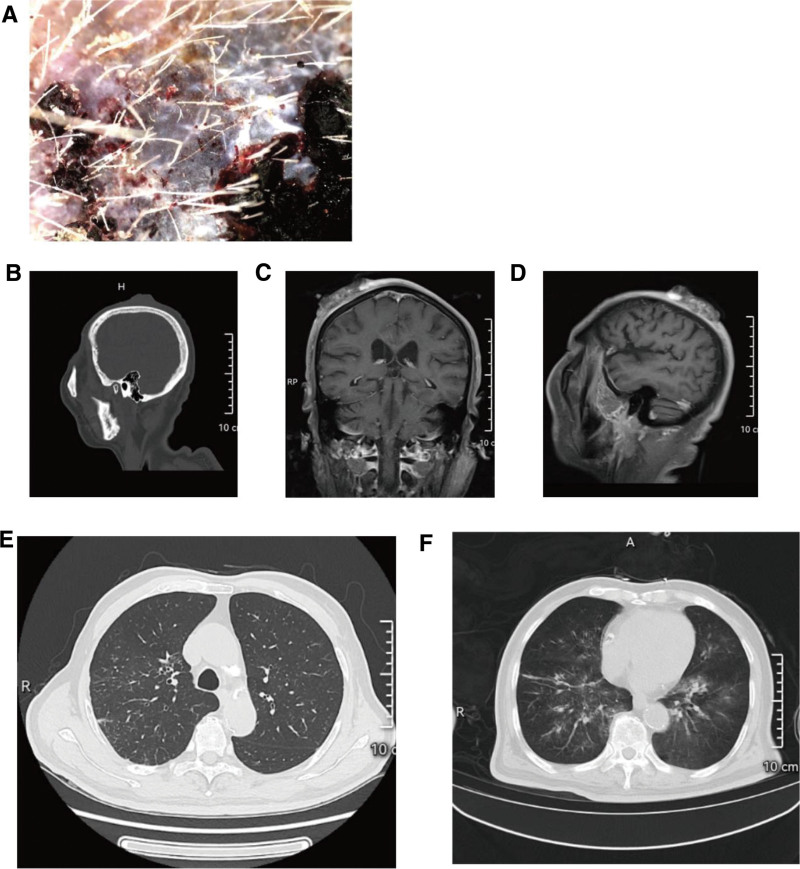
(A) Dermoscopic findings of the scalp mass of the patient on admission. (B) The patient’s head CT (sagittal view) at admission. (C) The patient’s head MRI (coronal view) at admission. (D) The patient’s head MRI (sagittal view) at admission. (E) Chest CT findings of the patient on admission. (F) Chest CT findings of the patient at the time of sudden massive hemoptysis, 5 months after surgery. CT = computed tomography, MRI = magnetic resonance imaging.

**Figure 3.**
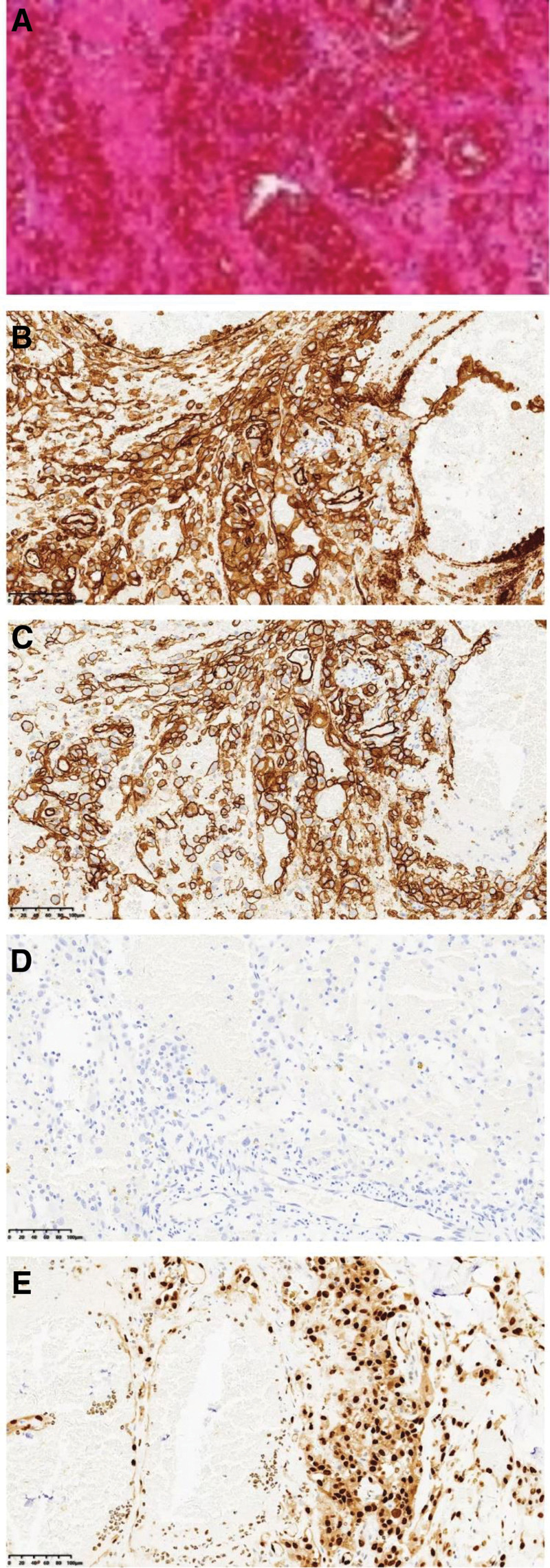
Partial immunohistochemical results of the patient’s scalp mass. (A) HE staining, 20X. (B) CD31 (+), 20X. (C) CD34(+), 20X. (D) CKP (+), 20X. (E) ERG (+), 20X.

White blood cell 5.5 × 10^9^/L, red blood cell 3.84 × 10^12^/L, hemoglobin 121g/L, platelet 188 × 10^9^/L. The electrocardiogram showed sinus rhythm. The electric axis deviated to the left. Dermoscopy showed that the lesions had irregular margins, an infiltrative growth pattern, erosions, ulcers, punctate hemorrhage, and massive blood vessels on the surface (Fig. 2A). B-ultrasound of the body mass showed a hypoechoic mass under the skin with an unclear boundary, and color Doppler flow imaging showed obvious blood flow signals in the mass. B-ultrasonography of the neck, axillary and groin did not show obvious enlarged lymph nodes. Cranial computed tomography (CT) showed a soft tissue protrusion on the top of the scalp, and no obvious abnormality was found in the skull (Fig. 2B). Contrast-enhanced magnetic resonance imaging of the head showed clumped long T1 and long T2 signal shadows on the right parietal scalp, and the enhancement showed obvious heterogeneous enhancement. There was no obvious bone destruction in the adjacent skull (Fig. 2C and D). Chest CT showed multiple small nodules with partial calcification in both lungs (Fig. 2E). Pathological examination of the nodules on the top of the head showed irregular vascular lumen formation in the dermis and obvious atypical endothelial cells, suggesting angiosarcoma (Fig. 3A). Immunohistochemical display CD31 (+), CD34 (+), ERG (+), human herpesvirus-8 (−), S-100 (−), and human melanoma black 45 (−) (Fig. 3B–E). In summary, the patient was diagnosed with angiosarcoma of the scalp.

### 2.2. Treatment

After consultation with the patient and his family, the basic condition of the patient was fully evaluated, and wide local excision of the scalp mass (including the erythema around the tumor) was performed under general anesthesia (Fig. 1C). The scalp was separated along the periosteal surface, the mass was cut intact, and the exposed area of the scalp was taken from the left thigh of the patient for skin grafting (Fig. 1D–F). Postoperative histopathology confirmed angiosarcoma, and the resection margin was negative. During the postoperative follow-up, partial skin necrosis and skull exposure were found in the scalp skin graft area (Fig. 1G and H). The patient refused further wound repair, positron emission tomography/computed tomography (PET-CT) and other examinations, radiotherapy, and chemotherapy. The patient was followed up until 5 months after the operation, and died due to sudden hemoptysis (Fig. 2F). The patient’s family gave consent for the publication of clinical and image details of the patient.

## 3. Discussion

Angiosarcoma is a rare and highly malignant endothelial cell tumor that often occurs in the head and neck of the elderly.^[1]^ A retrospective study in the United States found that more than 70% of angiosarcomas occur in the skin, subcutaneous, and breast vessels, and more than 20% of angiosarcomas occur in visceral sites or rare sites.^[11]^ The first symptom of scalp angiosarcoma was a painless scalp mass, ranging from 0.5 to 5 cm in diameter, with a soft texture and unclear boundary, some of which were accompanied by skin redness and elevated skin temperature. This can be followed by rapid enlargement of the mass, ulceration, bleeding, and pain. A small number of cases present with regional lymphadenopathy, mainly in the neck at the initial diagnosis.^[12]^ The early clinical manifestations are easily confused with benign lesions such as hemangioma, vascular malformation, seborrheic keratosis, or infectious lesions, and malignant tumors such as melanoma, resulting in difficulties in diagnosis.^[13–15]^ Head CT/magnetic resonance imaging showed irregular thickening or mass of scalp soft tissue with blurred boundaries, and the enhanced scan showed obvious uneven enhancement. Bone destruction of the skull can be seen in some cases. PET-CT may detect distant metastases such as lung, liver, and bone (about 30%–40% of the patients present at initial diagnosis or during follow-up).^[16]^ Dermoscopy can provide important clues for diagnosis, but the diagnosis still relies on histopathological examination and immunohistochemical results.^[16]^

At present, the treatment of scalp angiosarcoma is mainly based on surgical treatment, which mainly uses wide resection (the margin is 1–2 cm from the edge of the tumor), and partial skull resection combined with partial skull resection in cases of partial skull invasion, supplemented by radiotherapy, and chemotherapy may be used in high-risk cases (positive surgical margin and lymph node metastasis).^[17–21]^ The reconstruction of large scalp defects with sufficient margin is also a challenge, because the clinician must consider functional and aesthetic considerations, as well as prepare for postoperative radiotherapy.^[17,18]^ To compensate for the aesthetic effect, full-thickness skin grafting is the first choice.^[19]^ In addition, layered skin grafting, dermal regeneration template, dermal substitute, and acellular skin substitute Integra® can be applied to the reconstruction of scalp defects, and even to detect tumors.^[19,22–24]^ However, these options only play a replacement role after autologous skin graft necrosis, which needs to be fully evaluated by clinicians. Due to the large area of skin defect after the operation, measures to promote wound healing, promote graft survival, and reduce bacterial infection need to be carried out as soon as possible. Postoperative chemotherapy usually uses taxanes such as paclitaxel, anthracyclines such as doxorubicin, and isocyclic amides, which can reduce the risk of metastasis and death.^[25–27]^ However, due to the strong invasiveness and ill-defined boundary of angiosarcoma, complete resection is difficult, and the local recurrence rate is high. Doctors are also actively exploring other treatment methods.^[12]^ In recent years, there have been many studies on vascular endothelial growth factor receptors in the treatment of angiosarcoma. Although the related pathway activity is high, the drugs targeting vascular endothelial growth factor receptors, such as bevacizumab, have not shown ideal results in clinical trials.^[28,29]^ Immune checkpoint inhibitors, such as programmed cell death 1/programmed cell death ligand 1 inhibitors, have achieved some exciting efficacy in some patients with angiosarcoma, but their applicable populations and predictive markers still need to be further studied.^[30,31]^ The most common gene alterations in patients with head and neck angiosarcoma, such as *TP53*, *POT1*, and *CRKL*, are also common in other tumors, but still show certain specificity.^[32]^ The treatment of these altered gene pathways, combined with immunotherapy, may be more effective against tumors.^[33]^ Some case reports have shown that angiosarcoma responds to propranolol, a nonspecific β-adrenergic receptor antagonist, which is widely used in the treatment of hemangioma and has an inhibitory effect on animal angiosarcoma cell models.^[34,35]^ Studies in animal models have also found that the loss of some key tumor suppressor microRNAs in angiosarcoma and the activation of the mammalian target of rapamycin pathway promote the development of angiosarcoma.^[36,37]^

Angiosarcoma has a poor prognosis, with a 5-year survival rate of <30%. A meta-analysis found that the prognostic factors of angiosarcoma are mainly affected by age, tumor size, tumor location, resection margin status, lymph node or distant metastasis, and the integrity of the treatment plan (whether to standardize surgery and postoperative adjuvant therapy).^[38,39]^ Angiosarcoma most often metastasizes to the blood and is most likely to metastases to organs such as the lung, bone, and liver. Early metastasis is the main cause of death.^[38]^ Therefore, regular follow-up and PET-CT examination are important to evaluate whether patients have early metastasis. In this case, the age of the patient was over 70 years, the tumor size was more than 5 cm, and the tumor was located in the scalp. Although the surgical margin is negative, the treatment regimen is single, and the compliance is poor, which still has an adverse effect on the prognosis.

However, the current research on scalp angiosarcoma still has great limitations. The incidence of scalp vascular flesh is extremely low, and the clinical sample size is extremely scarce, which makes it difficult to carry out large sample prospective studies and insufficient statistical power.^[40]^ Pathological diagnosis is difficult, and it is easy to be confused with hemangioendothelioma, inflammatory granulation tissue, which may lead to misjudgment of the study subjects.^[41]^ The evidence base of treatment is weak, lacks unified standards, relies on case reports or small series of retrospective studies, and the verification of curative effect is limited.^[9]^ The basic research lags behind, the pathogenesis is unknown, and the lack of stable cell lines and animal models restricts the exploration of the mechanism. We also hope to establish an angiosarcoma multidisciplinary team, formulate a standard diagnosis and treatment process, establish a perfect biobank and clinical database of angiosarcoma, promote the construction of a regional vascular sarcoma research network, and explore precise treatment strategies based on molecular characteristics, so as to improve the prognosis of patients.

## 4. Conclusion

Scalp angiosarcoma is a rare but highly malignant tumor. Early diagnosis and comprehensive treatment are the keys to improving the prognosis. Clinicians should improve their understanding of the disease, combined with pathology, imaging, and immunohistochemistry to confirm the diagnosis, and adopt the treatment strategy of multidisciplinary collaboration, such as surgery, radiotherapy, and chemotherapy. For elderly patients, it is necessary to fully evaluate the risks and treatment benefits of surgery, and strengthen follow-up to detect metastases early and improve the quality of life of patients.

This work was supported by the second batch of major (key) science and technology plan projects in Jinhua (2022-3-101).

## Author contributions

**Conceptualization:** Xuefeng Fu, Yuxi Zhang.

**Data curation:** Xuefeng Fu, Lei Zeng, Qiying Zhang.

**Resources:** Lei Zeng.

**Project administration:** Qiying Zhang.

**Supervision:** Yuxi Zhang.

**Writing – original draft:** Yuxi Zhang.

**Writing – review & editing:** Yuxi Zhang.
